# Ethnic Differences in the Prevalence of Type 2 Diabetes Diagnoses in the UK: Cross-Sectional Analysis of the Health Improvement Network Primary Care Database

**DOI:** 10.2147/CLEP.S227621

**Published:** 2019-12-31

**Authors:** Tra My Pham, James R Carpenter, Tim P Morris, Manuj Sharma, Irene Petersen

**Affiliations:** 1 MRC Clinical Trials Unit at UCL, London WC1V 6LJ, UK; 2 Department of Primary Care and Population Health, University College London, London NW3 2PF, UK; 3 Department of Medical Statistics, London School of Hygiene & Tropical Medicines, London WC1E 7HT, UK; 4 Department of Clinical Epidemiology, Aarhus University, Aarhus N 8200, Denmark

**Keywords:** ethnicity, type 2 diabetes, primary care database, electronic health records, multiple imputation, missing not at random

## Abstract

**Aims/Hypothesis:**

Type 2 diabetes mellitus is associated with high levels of disease burden, including increased mortality risk and significant long-term morbidity. The prevalence of diabetes differs substantially among ethnic groups. We examined the prevalence of type 2 diabetes diagnoses in the UK primary care setting.

**Methods:**

We analysed data from 404,318 individuals in The Health Improvement Network database, aged 0–99 years and permanently registered with general practices in London. The association between ethnicity and the prevalence of type 2 diabetes diagnoses in 2013 was estimated using a logistic regression model, adjusting for effect of age group, sex, and social deprivation. A multiple imputation approach utilising population-level information about ethnicity from the UK census was used for imputing missing data.

**Results:**

Compared with those of White ethnicity (5.04%, 95% CI 4.95 to 5.13), the crude percentage prevalence of type 2 diabetes was higher in the Asian (7.69%, 95% CI 7.46 to 7.92) and Black (5.58%, 95% CI 5.35 to 5.81) ethnic groups, while lower in the Mixed/Other group (3.42%, 95% CI 3.19 to 3.66). After adjusting for differences in age group, sex, and social deprivation, all minority ethnic groups were more likely to have a diagnosis of type 2 diabetes compared with the White group (OR Asian versus White 2.36, 95% CI 2.26 to 2.47; OR Black versus White 1.65, 95% CI 1.56 to 1.73; OR Mixed/Other versus White 1.17, 95% CI 1.08 to 1.27).

**Conclusion:**

The prevalence of type 2 diabetes was higher in the Asian and Black ethnic groups, compared with the White group. Accurate estimates of ethnic prevalence of type 2 diabetes based on large datasets are important for facilitating appropriate allocation of public health resources, and for allowing population-level research to be undertaken examining disease trajectories among minority ethnic groups, that might help reduce inequalities.

## Introduction

Type 2 diabetes mellitus is associated with substantial disease burden, including increased mortality risk and significant long-term morbidity.^1^ The global prevalence of diabetes in adults has increased considerably over the last decade; from 30 million in 1964 to more than 400 million in 2015, equivalent to 8.8% of the population aged between 20 and 79 years.^2^ However, there are substantial differences in the prevalence of diabetes at regional level, and in particular among different ethnic groups.^2^–^9^ There is widespread acceptance that the prevalence of type 2 diabetes is indeed higher among Asian, Black and minority ethnic (BME) groups in the UK.^10^ However, there are limited data available, and the last large-scale survey was conducted in the early 2000s. The 2004 Health Survey for England (HSfE) collected data from around 13,500 adults and suggested that the prevalence of type 2 diabetes was much higher in Black Caribbean (9.5% men, 7.6% women), Indian (9.2% men, 5.9% women), Pakistani (7.3% men, 8.4% women), and Bangladeshi (8.0% men, 4.5% women) than in the general population (3.8% men, 3.1% women).^5^

Despite significant advances in the management of type 2 diabetes in recent years, newer estimates regarding prevalence of the disease in different ethnic groups in the UK setting remain limited.^10^ This has been hampered by limited datasets available detailing ethnicity, and an inability to handle some of the challenges posed by data quality when ethnicity information isavailable.^11^,^12^ However, understanding disease patterns in minority ethnic groups is important for population-based diabetes screening, designing lifestyle interventions, and epidemiological research.^11^

Opportunities to undertake more ethnicity-related research has arisen from the gradual shift in the management of type 2 diabetes from hospitals towards primary care.^13^ This provides potential for studying the association between ethnicity and type 2 diabetes on a large scale using readily available primary care data.^7^,^14^ Additionally, since the introduction of the National Health Service (NHS)’s Quality and Outcomes Framework (QOF)^15^ in 2004, general practitioners have been offered financial incentives for monitoring and managing chronic diseases in primary care including diabetes, hence data quality has improved. Growing recognition of ethnicity as a risk factor for several common long-term illnesses has also led to considerable improvement in the recording of ethnicity in general practice records.^16^

In this study, we examined the prevalence of type 2 diabetes diagnoses based on primary care electronic health records of individuals who were registered with general practices in London, one of the most ethnically diverse regions in the UK.

## Methods

### Data Source

We analysed electronic health records data from The Health Improvement Network (THIN)^17^ primary care database. The database contains longitudinal records of patients’ medical conditions, symptoms, diagnoses, and medications prescribed during consultations in primary care, from the time the patients register with the general practices to when they leave or die. Clinical information including symptoms and diagnoses are recorded using Read codes, a hierarchical coding system.^18^ THIN also holds information on patient demographic characteristics, such as sex and year of birth. In addition, social deprivation status is measured by quintiles of the Townsend deprivation score,^19^ a composite index of occupation, car ownership, overcrowding, and unemployment, based on the individuals’ postcode and information from the 2001 census data. The database has been used in previous studies on type 2 diabetes.^7^,^14^

### Ethical Approval

Use of THIN for scientific research was approved by the National Health Service South‐East Multicentre Research Ethics Committee in 2003. Scientific approval to undertake this study was obtained from IQVIA World Publications Scientific Review Committee in September 2017 (reference number 17THIN083).

### Study Sample

We included individuals who were permanently registered with general practices in London and contributing data to THIN. This sample was chosen due to the high level of ethnic diversity in London, and was thus relevant for the study of ethnic differences in the prevalence of type 2 diabetes diagnoses.

Individuals were selected into the study sample if they were actively registered with general practices located in London and contributing data to THIN on 01 January 2013. Individuals were also required to have been registered with the same practices for at least 12 months by this date, to allow enough time for their type 2 diabetes status to be recorded in the electronic health records. For quality assurance, we included only data from practices where there was evidence that they were fully computerised and their mortality recording was on par with the data provided from the Office for National Statistics (ONS).^20^,^21^

### Outcome and Explanatory Variables

The recording of diabetes diagnoses and management in THIN is comprehensive and therefore there are several ways an individual may be identified as diabetic. We used an algorithm developed by Sharma et al to identify individuals with type 2 diabetes.^14^ This algorithm identifies individuals as having diabetes if they have at least two of the following records: a diagnostic code for diabetes, supporting evidence of diabetes (e.g. screening for diabetic retinopathy), or a prescribed treatment for diabetes. We considered the first record of any of these three as the date of diagnosis of diabetes. We defined prevalent cases of type 2 diabetes as individuals who had a diagnosis of type 2 diabetes on or before 01 January 2013.

Information on ethnicity is typically recorded in THIN using Read codes.^22^ A Read code list including codes related to ethnicity was developed using a previously published method.^18^,^23^ The majority of the identified ethnicity records were found by searching the medical and additional health data files for Read codes in the ethnicity code list. We obtained limited additional information from the pre-anonymised free text and other free text linked to ethnicity-related Read codes. Ethnicity information was then coded into the White, Mixed, Asian, Black, and Other ethnic groups, in line with the five-level ONS categorisation.^24^ The Mixed and Other groups were then combined due to their small counts and heterogeneity.

### Statistical Analysis

We examined the association between ethnicity and the prevalence of type 2 diabetes diagnoses in THIN using a logistic regression model. The outcome variable was a binary indicator of whether an individual had a diagnosis of type 2 diabetes on or before 01 January 2013. Covariates in the model included the individual’s ethnic group (defined as White, Asian, Black, Mixed/Other), age, sex, and social deprivation status (defined in quintiles of the Townsend score).^19^ Age was analysed in 10-year age groups for individuals aged 0–79 years, together with an 80+ group for those who were ≥80 years old. Individuals with incomplete information on age, sex, and deprivation status were excluded from the analysis, leaving missing data only in ethnicity.

Missing values in ethnicity were handled by calibrated-δ adjustment multiple imputation.^25^ This method utilises UK census information about the population-level distribution of ethnicity to impute missing values under a missing not at random assumption.^26^ Calibrated-δ adjustment multiple imputation helps overcome the limitations of standard multiple imputation in the setting where the completeness of ethnicity information in primary care may be differential across ethnic groups, even after controlling for other factors associated with the recording of ethnicity in the analysis. Further details on how multiple imputation of missing values in ethnicity was performed are presented in Sections S1–S2 and Tables S1–S2, Supplementary materials.

In our paper previously published in Statistics in Medicine,^25^ we included a simplified version of the analysis reported here as a case study for the purpose of demonstrating a new multiple imputation method (calibrated-δ adjustment multiple imputation). This analysis represents our attempt to address the clinical question, in which missing values in ethnicity were imputed from a more complex model.

## Results

### Characteristics of Study Sample

**Figure 1:**
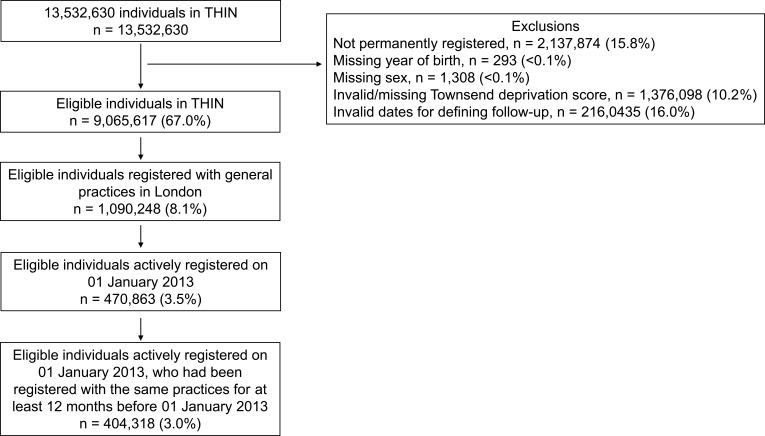
Flowchart of the inclusion criteria for study sample. **Note:** Adapted from Pham TM, Carpenter JR, Morris TP, Wood AM, Petersen I. Population-calibrated multiple imputation for a binary/categorical covariate in categorical regression models. Stat Med. 2019;38(5):792–808. doi:10.1002/sim.8004. Creative Commons license and disclaimer available from: http://creativecommons.org/licenses/by/4.0/legalcode.^25^ **Abbreviation:** THIN, The Health Improvement Network.

We identified 404,318 individuals who were actively registered with general practices in London on 01 January 2013 (Figure 1, Table 1). These individuals had been registered with the same practices for at least 12 months by this date.

**Table 1:** Summary of Demographic Characteristic and Disease Variables, N=404,318

Variable	n	%
**Sex**		
Men	198,301	49
Women	206,017	51
**Age Group (Years)**		
0–9	41,601	10.3
10–19	45,664	11.3
20–29	50,065	12.4
30–39	65,695	16.2
40–49	64,837	16
50–59	53,272	13.2
60–69	39,427	9.8
70–79	25,348	6.3
80+	18,409	4.5
**Townsend Deprivation Score**		
Quintile 1 (least deprived)	48,934	12.1
Quintile 2	64,788	16
Quintile 3	101,305	25.1
Quintile 4	102,626	25.4
Quintile 5 (most deprived)	86,665	21.4
**Ethnic Group**		
White	224,403	55.5
Asian	35,027	8.7
Black	30,771	7.6
Mixed/Other	19,483	4.8
Missing	94,634	23.4
**Disease Indicator**		
Type 2 diabetes	22,100	5.5
Heart attack	5,101	1.3
Stroke	7,670	1.9
Chronic kidney disease	18,584	4.6
Sickle cell disease	311	0.1
Thalassaemia	2,282	0.6
Schizophrenia	2,059	0.5
**Total**	404,318	100

**Note:** Adapted from Pham TM, Carpenter JR, Morris TP, Wood AM, Petersen I. Population-calibrated multiple imputation for a binary/categorical covariate in categorical regression models. Stat Med. 2019;38(5):792-808. doi:10.1002/sim.8004. Creative Commons license and disclaimer available from: http://creativecommons.org/licenses/by/4.0/legalcode.^25^

The sample comprised 51% women; the majority of individuals in the sample (approximately 80%) were below 60 years of age; there were slightly more than 70% of the individuals with quintiles of Townsend score ≥3; and 5.5% (22,100) of the individuals had a diagnosis of type 2 diabetes on or before 01 January 2013.

Ethnicity was available for 309,684 (76.6%) and missing for 94,634 (23.4%) individuals (Table 1). The observed distribution of ethnicity (via a complete record analysis) showed an overestimation of the White ethnic group and an underestimation of the Asian and BME groups, compared with the census distribution (Figure S1, Supplementary materials). On the other hand, calibrated-δ adjustment multiple imputation recovered the census distribution of ethnicity in the imputed data (Figure S1, Supplementary materials).

### Association Between Ethnicity and the Prevalence of Type 2 Diabetes Diagnoses

Compared with the White ethnic group (5.04%, 95% CI 4.95 to 5.13), the crude percentage prevalence of type 2 diabetes was higher in the Asian (7.69%, 95% CI 7.46 to 7.92) and Black (5.58%, 95% CI 5.35 to 5.81) groups, while lower in the Mixed/Other group (3.42%, 95% CI 3.19 to 3.66) (Table 2).

**Table 2:** Prevalence of Type 2 Diabetes Diagnoses by Socio-Demographic Factors Under Calibrated-δ Adjustment Multiple Imputation, N=404,318, M=30 Imputations

Variable	Crude Prevalence (%)^a^	95% CI	Adjusted OR^b^	95% CI
**Ethnic Group**				
White	5.04	4.95 to 5.13	1	
Asian	7.69	7.46 to 7.92	2.36	2.26 to 2.47
Black	5.58	5.35 to 5.81	1.65	1.56 to 1.73
Mixed/Other	3.42	3.19 to 3.66	1.17	1.08 to 1.27
**Sex**				
Men	5.88	5.78 to 5.98	1	
Women	5.07	4.97 to 5.16	0.77	0.75 to 0.8
**Age Group (Years)**				
0–9	0.04	0.02 to 0.06	0.01	0.01 to 0.02
10–19	0.10	0.07 to 0.13	0.03	0.02 to 0.03
20–29	0.49	0.43 to 0.55	0.12	0.11 to 0.14
30–39	1.32	1.23 to 1.40	0.33	0.31 to 0.36
40–49	3.69	3.55 to 3.84	1	
50–59	8.42	8.18 to 8.65	2.52	2.39 to 2.65
60–69	14.26	13.91 to 14.60	4.93	4.69 to 5.19
70–79	19.70	19.21 to 20.19	7.49	7.11 to 7.89
80+	18.64	18.08 to 19.20	7.62	7.19 to 8.06
**Townsend Deprivation Score**				
Quintile 1 (least deprived)	4.97	4.78 to 5.16	1	
Quintile 2	5.15	4.98 to 5.32	1.12	1.06 to 1.18
Quintile 3	5.36	5.22 to 5.50	1.25	1.19 to 1.31
Quintile 4	5.42	5.28 to 5.56	1.47	1.40 to 1.55
Quintile 5 (most deprived)	6.16	6.00 to 6.32	1.86	1.77 to 1.96

**Notes:** ^a^Unadjusted percentage prevalence of type 2 diabetes diagnoses by ethnic group, sex, age group and deprivation status. ^b^OR: odds ratios of having a type 2 diabetes diagnosis among the Black and minority ethnic groups compared to the White ethnic group, adjusted for sex, age group and Townsend deprivation score in a multivariable logistic regression model.

**Figure 2:**
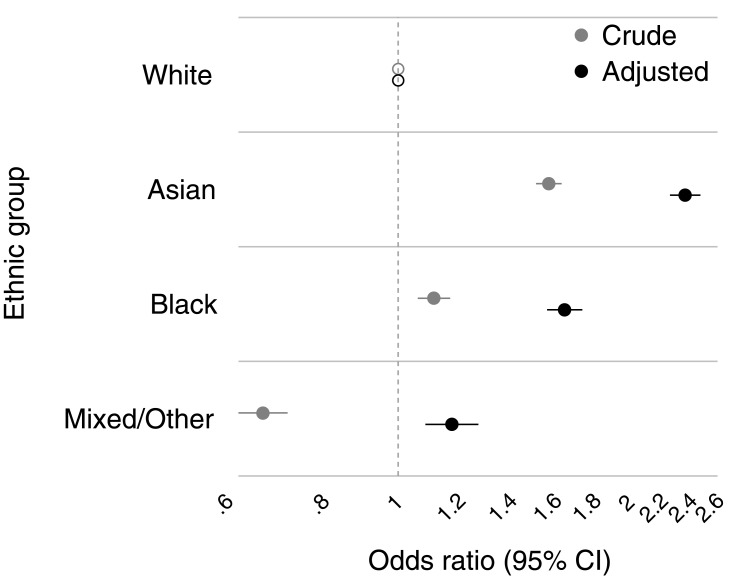
Association between type 2 diabetes diagnosis and ethnic group, both crude (grey circles) and adjusted for sex, age group, and deprivation status (black circles) under calibrated-δ adjustment multiple imputation, n=404,318, m=30 imputations. Hollow circles: the White ethnic group was set as the reference category for ethnicity. **Abbreviation:** CI, confidence interval.

Table 2 and Figure 2 present the odds ratios (OR) and 95% confidence intervals (CI) for the association between ethnicity and the prevalence of type 2 diabetes diagnoses, adjusted for age group, sex, and social deprivation, under calibrated-δ adjustment multiple imputation. Overall, the Asian and Black ethnic groups were more likely to have a type 2 diabetes diagnosis compared with the White group (Figure 2), after adjustment for age, sex, and social deprivation (OR Asian versus White 2.36, 95% CI 2.26 to 2.47; OR Black versus White 1.65, 95% CI 1.56 to 1.73). The odds of being diagnosed with type 2 diabetes were lower in women compared with men (OR 0.77, 95% CI 0.75 to 0.80), and increased smoothly with older age groups (OR 60–69 years versus 40–49 years 4.93, 95% CI 4.69 to 5.19) and more deprived quintiles of the Townsend score (OR quintile 5 (most deprived) versus quintile 1 (least deprived) 1.86, 95% CI 1.77 to 1.96).

Similar patterns were seen after missing values in ethnicity had been handled by a complete record analysis, single imputation with the White ethnic group, and standard (uncalibrated) multiple imputation, although the estimated ORs for the Asian and BME groups were higher compared with our primary method calibrated-δ adjustment multiple imputation (Table S3, Supplementary materials).

## Discussion

### Summary of Results

Our study used data from a large UK primary care database to estimate the ethnic prevalence of type 2 diabetes in primary care. Compared with those of White ethnicity, the likelihood of having a type 2 diabetes diagnosis was more than double among Asian people, 65% more likely among Black people, and 17% more likely among people of Mixed/Other ethnicities, after adjustment for other demographic characteristics. Using ONS census data, we were able to impute missing data related to ethnicity and calibrate our multiple imputation model such that the ethnic distribution in our imputed datasets matched that of the general population in London.

### Findings in Relation to Other Evidence and Implications

Data from the 2004 HSfE remain the most commonly cited sources of ethnic prevalence in the UK. However, it did not adjust for differences in prevalence driven by several demographic factors and social deprivation as in this study, which are both independent risk factors in their own right for the disease. Therefore, though we cannot compare our results directly to the findings from the HSfE, we note that our crude prevalence estimates of type 2 diabetes were similar for the Asian ethnic group. The prevalence of type 2 diabetes appeared to be lower among Black people in our study than among Black Caribbean in the HSfE.^5^ This might be because our sample includes individuals from both Black Caribbean and Black African and the prevalence differs between the two groups.

Analysis of data from the London Southall and Brent Revisited (SABRE) multi-ethnic cohort estimated that by the age of 80 years, 40–50% of South Asian and African-Caribbean men and women will have type 2 diabetes, at least twice the proportion of their age-matched cohort in a sample of 4,202 individuals.^27^ In our study, we also found that after adjusting for age, sex and social deprivation, Asian people were twice as likely to have a diagnosis of type 2 diabetes while those of Black ethnicity were 65% more likely, based on a highly diverse urban population of 404,318.

Most studies undertaken have reported higher prevalence in BME groups for type 2 diabetes; however, like the 2004 HSfE, studies thus far have generally been modest in sample size, unable to adjust for important demographic factors and social deprivation, while only a few have used imputation techniques to adequately account for missing data. Some previously reported estimates suggest that prevalence is nearly 3–5 times higher in BME groups,^10^ but our study highlights that when other demographic and socio-economic factors are accounted for, relative prevalence is unlikely to be as high as this. Nevertheless, our finding that the likelihood of diagnosis being over double that in Asian people and over 65% more likely in Black people still highlights major ethnic inequalities when compared with the White British population.

Identifying prevalence patterns in ethnicity for type 2 diabetes accurately is important as it can help ensure appropriate allocation of public health resources for diabetes screening and lifestyle interventions which often need to be tailored for different ethnicities.^11^ Recent work has suggested that onset of diabetes in the Asian and Black populations may be up to 12 years earlier on average.^28^ Understanding ethnic prevalence also allows for further work to be conducted at the population level, examining, for example, ethnic variation in response to different pharmacotherapies which has been reported previously.^29^ It is increasingly recognised that patterns of diabetic complications may also be distinct among BME groups,^30^–^32^ with higher reported rates of nephropathy among Asian people in particular. Previous studies investigating this association have been impeded by the need to use complete record analysis due to missing data in ethnicity, which can create systematic bias in estimates.^33^ The use of our approach would help overcome this limitation.

## Strengths and Limitations

This study was based on the analysis of a large sample of individuals in primary care, allowing us to adequately adjust for age, sex and social deprivation, which are independent risk factors of type 2 diabetes.^7^

Using the calibrated-δ adjustment multiple imputation method for handling missing data in ethnicity, we were able to incorporate the census data in the imputation process, thus recovering the census distribution of ethnicity in the imputed data and calibrating our inference to the population level.

Some limitations of our study warrant consideration. The small counts in many of the ethnic groups prevented us from further categorising the recorded ethnicity information in THIN into the 16-level (minor) ONS classification. Previous work comparing the level of discordance between hospital-recorded ethnicity in the Hospital Episode Statistics database and self-reported ethnicity in a large cancer patient survey suggested that a broader classification of routinely collected ethnicity data is more reliable.^34^ In addition, multiple imputation of ethnicity based on the 16-level classification may be problematic and is likely to be inaccurate.

In our analysis, we were not able to account for factors such as education and physical activity level (such information is not consistently recorded in primary care electronic health records), as well as other lifestyle health indicators such as body mass index and smoking status (which also contain missing values). This is partly due to the constraint that, at presence, the calibrated-δ adjustment multiple imputation method has only been developed and evaluated for handling missing values in a single variable. Similarly, we could not exclude the possibility of other omitted confounders. However, we were able to control for several important diabetes risk factors (including age, sex, social deprivation), which is an improvement from several previous work.

Our results provided estimates for the prevalence of type 2 diabetes diagnoses in the primary care setting, which might not fully reflect the true extent of how prevalent the condition remains in the overall population. This is due to the existence of a population of diabetic individuals who remain undiagnosed. Indeed, according to Goff,^10^ while 5.6% of the population have a diagnosis of diabetes, the true prevalence might be close to 7.4%.

## Conclusion

In conclusion, after accounting for age, sex, and social deprivation status, our results indicated that compared with the White ethnic group, the likelihood of having a type 2 diabetes diagnosis was more than double among the Asian ethnic group, and also elevated by 65% among the Black group and by 17% among the Mixed/Other group. Accurate estimates of ethnic prevalence of type 2 diabetes are important for ensuring public health resources are allocated appropriately for diabetes screening and lifestyle interventions. These estimates also provide the basis for more precise large-scale population-level research to be undertaken, examining diabetes disease trajectories and complications among BME groups, which would help identify, and potentially reduce health inequalities.
